# Effect of a 6-week dynamic neuromuscular training programme on ankle joint function: A Case report

**DOI:** 10.1186/1758-2555-3-13

**Published:** 2011-06-09

**Authors:** Jeremiah O'Driscoll, Fearghal Kerin, Eamonn Delahunt

**Affiliations:** 1 Mount Carmel Hospital, Dublin, Ireland; 2 School of Public Health, Physiotherapy and Population Science, University College Dublin, Dublin, Ireland; 3 Institute for Sport and Health, University College Dublin, Dublin, Ireland

**Keywords:** ankle sprain, ankle instability, ankle injury, rehabilitation, injury prevention

## Abstract

**Background:**

Ankle joint sprain and the subsequent development of chronic ankle instability (CAI) are commonly encountered by clinicians involved in the treatment and rehabilitation of musculoskeletal injuries. It has recently been advocated that ankle joint post-sprain rehabilitation protocols should incorporate dynamic neuromuscular training to enhance ankle joint sensorimotor capabilities. To date no studies have reported on the effects of dynamic neuromuscular training on ankle joint positioning during landing from a jump, which has been reported as one of the primary injury mechanisms for ankle joint sprain. This case report details the effects of a 6-week dynamic neuromuscular training programme on ankle joint function in an athlete with CAI.

**Methods:**

The athlete took part in a progressive 6-week dynamic neuromuscular training programme which incorporated postural stability, strengthening, plyometric, and speed/agility drills. The outcome measures chosen to assess for interventional efficacy were: [1] Cumberland Ankle Instability Tool (CAIT) scores, [2] Star Excursion Balance Test (SEBT) reach distances, [3] ankle joint plantar flexion during drop landing and drop vertical jumping, and [4] ground reaction forces (GRFs) during walking.

**Results:**

CAIT and SEBT scores improved following participation in the programme. The angle of ankle joint plantar flexion decreased at the point of initial contact during the drop landing and drop vertical jumping tasks, indicating that the ankle joint was in a less vulnerable position upon landing following participation in the programme. Furthermore, GRFs were reduced whilst walking post-intervention.

**Conclusions:**

The 6-week dynamic neuromuscular training programme improved parameters of ankle joint sensorimotor control in an athlete with CAI. Further research is now required in a larger cohort of subjects to determine the effects of neuromuscular training on ankle joint injury risk factors.

## Introduction

The human ankle joint is one of the most frequently injured joints in the human body, with lateral ligament sprains being the most common type of ankle injury [1]. Following initial sprain the development of residual symptoms are not uncommon [2]. Two of the most frequently encountered residual symptoms include feelings of ankle joint instability, and reported episodes of "giving way". Subjects who describe the presence of both of these symptoms are considered to have functional instability (FI) [3]. Two other frequently used terms include mechanical instability (MI) of the ankle joint and chronic ankle instability (CAI). MI of the ankle joint refers to the presence of increased rear-foot inversion laxity or excessive anterior talocrural excursion, while CAI is used as an all encompassing term to indicate the presence of both FI and MI [3].

The treatment and rehabilitation of individuals with CAI poses a significant challenge for clinicians, and has enormous healthcare and economic costs [4]. A number of studies have investigated the effect of various rehabilitation protocols on ankle joint sensorimotor control [5-9]. Observed effects include enhanced strength [9], heightened joint position sense [7,8] and improved postural stability [6,7]. Furthermore, in two of these studies ankle joint function as measured by the Functional Ankle Disability Index (FADI) also improved following completion of the rehabilitation protocol, which is indicative of improved function and decreased disability [6,7].

One of the primary mechanisms of ankle joint sprain involves aberrant ankle joint positioning upon initial contact with the ground during the transition from an unloaded to a loaded state (i.e. on initial contact upon landing from a jump or initial contact when walking or running) [10]. However to date no studies have examined the effect of neuromuscular training on ankle joint positioning at initial contact during walking and jump landing. It has recently been advocated that studies should examine the effects of dynamic neuromuscular training protocols on established ankle joint injury risk factors and sensorimotor control [11].

Thus the objective of the present study was to examine the effect of a 6-week dynamic neuromuscular training programme on parameters of sensorimotor function in an athlete with chronic ankle instability (CAI).

## Methods

### Subject

The 19-year-old athlete (caucasian male, height 1.83 m, body mass 83 kg) was injured whilst competing in a line-out during an amateur rugby union game, in January 2008. The mechanism of injury involved a "forced inversion" of the left ankle joint as a result of landing awkwardly from the line-out jump. The athlete was unable to continue participation in the game and that evening attended the Accident and Emergency Department of a nearby hospital. X-ray imaging failed to reveal any bony abnormality. The ankle joint was markedly ecchymotic and edematous and the athlete was prescribed simple analgesics and given crutches to use for mobilization. He was advised to progress from non-weight bearing (NWB) to full-weight bearing (FWB) and normal function as pain permitted.

Following the initial injury the athlete who himself was an under-graduate physiotherapy student consulted one of the authors (ED). On examination mechanical instability was evident upon anterior drawer testing. Initially it was decided to institute a course of manual therapy. Anterior-posterior (AP) mobilisations of the inferior tibiofibular and talocrural joints were performed once a week for a period of six weeks in keeping with positional faults noted on assessment (i.e. restriction in weight-bearing dorsiflexion and reduced posterior glide of the fibula at the inferior tibiofibular joint). In conjunction with this manual therapy the athlete was prescribed some simple static postural stability exercises. These included single leg stance exercises with eyes open and eyes closed. Following this course of treatment the athlete was fully weight-bearing and the swelling had largely resolved.

Despite these measures, six months following the initial injury the athlete continued to complain of limitations during functional activities which were adversely affecting his level of sports participation. He described how he felt that he was "struggling" to reach the same level of sporting performance compared to his pre-injury status. Furthermore the athlete complained of recurrent episodes of his ankle joint "giving way" with increasingly innocuous activities, and a generalized feeling of ankle joint "weakness". During this six month period the athlete also reported one other significant inversion injury which required a short period of NWB.

At this time it was decided to undertake further radiological investigation. Magnetic resonance imaging (MRI) revealed fluid in the peroneal sheath suggestive of tenosynovitis, as well as thickening of the ATFL with some discontinuity of fibres at the fibular attachment in keeping with a grade 2 tear. At this stage the athlete decided to withdraw from formal sports participation primarily due to academic commitments.

The athlete did however occasionally participate in recreational sporting activities but again was limited due to regular episodes of ankle joint "rolling". In October 2009 the opportunity arose to enroll the athlete who was now in his final year as an under-graduate physiotherapy student in a dynamic neuromuscular training programme. This opportunity arose as the primary author (JOD) was due to undertake a research project for the part fulfillment of his M.Sc. degree, and subsequently chose to design a case study to investigate the effect of a dynamic neuromuscular training programme on ankle joint function in a subject with CAI. Full approval for the study was received by the University Human Research Ethics Committee and informed subject consent was acquired. The subject had no previous history of lower limb injury nor did he suffer with diabetes, peripheral neuropathy or any other condition known to effect postural stability or proprioception.

The novelty of this case is that it is the first to report on the effects of a dynamic neuromuscular training programme on ankle joint positioning during jump landing in a subject with CAI. In addition to measures of ankle joint kinematics during jump landing we also investigated the effects of the intervention on other important aspects of ankle joint function, such as walking kinetics, postural stability as well as self-reported ankle joint function as measured by the CAIT.

### Outcome Measures

#### Cumberland Ankle Instability Tool (CAIT)

The severity of ankle joint instability was initially assessed using the CAIT [12]. Prior to the initiation of the dynamic neuromuscular training programme the athlete had a CAIT score of 4 on the injured side. A score of ≤ 24 on the CAIT indicates the presence of FI. The athlete reported no previous history of injury on the uninvolved side and thus scored 30 on the CAIT.

#### Star Excursion Balance Test (SEBT)

Deficiencies in postural stability have been shown through prospective research to be a predictor of ankle joint injury development [13,14]. Furthermore a recently published meta-analysis has shown that subjects with CAI exhibit deficiencies in both static and dynamic postural stability [15].

One measure of dynamic postural stability commonly employed by clinicians and researchers alike is the SEBT. Previous research has shown that subjects with CAI have decreased performance on the SEBT as measured by reach distance, compared to non-injured controls [16,17]. During the performance of the SEBT, only the anterior, posterior-medial and posterior-lateral directions were used. SEBT performance was carried out in accordance with the methods published by Hertel et al [16]. Performance of the SEBT was conducted in a similar manner to those methods described in Delahunt et al [18]. The subject performed 3 reaches in each of the anterior, posterior-medial and posterior-lateral directions. The average value of the 3 reaches in each direction was used for further analysis. Quantification of reach distances were calculated in accordance with the work of Gribble et al [19]. Reach distances were divided by leg length and multiplied by 100 to calculate a dependent variable that represents reach distance as a percentage of leg length.

#### Drop landing

Previous research has suggested that an increased plantar flexion touch-down angle upon landing from a jump could precipitate the onset of an ankle joint sprain [10]. Thus, ankle joint sagittal plane angular displacement was measured by 3 CODA mpx1 motion analysis units with a sampling frequency of 200 Hz, with the particular lower limb set-up being previously described by Delahunt et al [20-22]. Ground reaction forces were recorded at 1000 Hz from a walkway embedded force-plate (AMTI, Watertown, MA, USA). We chose to examine the angle of ankle joint plantar flexion at the point of initial contact (IC) with the ground during a drop landing as well as the angle of ankle joint plantar flexion at the point of second IC with the ground during a drop vertical jump. The second IC with the ground during a drop vertical jump replicates loading of the limb akin to sporting participation, whereby a participant lands from a vertical jump having propelled oneself off the support surface. IC was defined as the point when the vertical component of ground reaction force exceeded 10 N. For both the drop landing and the drop vertical jump the participant initially stood on a 35 cm high platform.

During performance of the drop landing the participant initially stood on the non-injured leg and was required to "step" off the 35 cm high platform and land on the centre of the force-plate on the injured leg. This methodology has previously been described by Delahunt et al [22]. For the performance of the drop vertical jump the participant was required to initially perform a double leg landing on two adjacent force-plates and then propel himself immediately in a vertical trajectory akin to "jumping for a ball" similar to the methods described by Hewett et al [23]. The athlete performed 3 trials of both the drop landing and drop vertical jump in a counterbalanced sequence. The average value of the 3 trials was then utilized for subsequent analysis to investigate the difference between the pre- and post-intervention angle of plantar flexion.

#### Walking

Previous research has suggested that the gait profile of subjects with CAI differs from non-injured control subjects [21,24-26]. Specifically, subjects with CAI have been shown to exhibit a more inverted position of the rear-foot during the initial contact and loading phases of the stance phase, which the authors hypothesized could pre-dispose to repeated inversion injury [21,26].

Equally, subjects with CAI have also been shown to have a dorsiflexion deficit and altered shank-rearfoot coupling [24,25]. Recent evidence supports the idea that altered joint mechanics and in particular aberrant kinetics and kinematics could predispose to progressive cartilaginous micro-damage and the early onset of osteoarthritis [27].

We chose to investigate the rearfoot angular displacement at the point of IC with the force-plate during walking as well as the peak ground reaction force components during the stance phase. IC was defined as the point when the vertical component of ground reaction force exceeded 10 N. Lower limb joint angular displacements were captured at 200 Hz by 3 CODA mpx1 motion analysis units, while ground reaction forces were captured at 1000 Hz with a walkway embedded force-plate (AMTI, Watertown, MA, USA). The athlete performed 10 gait trials at self-selected walking pace with the average value of the ten trials being utilized for the analysis of differences between the pre- and post-intervention conditions.

### Standardized mean difference

A modified standardized mean difference (SMD) was calculated for the outcome measures as follows: *SMD = difference in mean outcome between groups/mean of the SD of the pre- and post-intervention outcomes*

#### Treatment/Intervention

The athlete took part in a progressive 6-week dynamic neuromuscular training programme incorporating postural stability, strength, plyometric, and speed/agility drills (Table 1).

**Table 1:** 6-week rehabilitation programme

	Week 1	Week 2	Week 3	Week 4	Week 5	Week 6
**Postural stability**	Single leg stance on Airex^® ^cushion: (*3 minutes)*	Single leg stance on tilt board: (*3 minutes*)	Single leg stance on BOSU^® ^ball: (*3 minutes)*	Single leg stance on BOSU^® ^ball with rebounding ball catches: (*3 minutes)*	Anterior jump lands from Reebox^® ^step: *(2 sets × 10 reps with 10 second stabilization)*	Lateral jump lands from Reebox^® ^step*(2 sets × 10 reps with 10 second stabilization)*
**Strength**	Double leg heel raises: *(3 sets × 12 reps)* Double leg bridge: *(2 sets × 10 reps)* Clam-shell gluteus medius: *(2 sets × 10 reps - each side)*	Double leg heel raises: *(3 sets × 12 reps)* Double leg bridge: *(2 sets × 10 reps)* Clam-shell gluteus medius: *(2 sets × 10 reps - each side)*	Single leg heel raises: *(2 sets × 10 reps - each side)* Single leg bridge: *(3 sets × 12 reps- each side)* Figure-4 gluteus medius: *(2 sets × 10 reps - each side)*	Single leg heel raises: *(2 sets × 10 reps - each side)* Single leg bridge: *(3 sets × 12 reps- each side)* Figure-4 gluteus medius: *(2 sets × 10 reps - each side)*	Single leg heel raises with weight (15 kg): *(3 sets × 12 reps - each side)* Double leg squats: *(3 sets × 12 reps)* Resisted lateral side-steps: *(3 sets × 12 reps/step - each sides)*	Single leg heel raises with weight (20 kg): *(3 sets × 12 reps - each side)* Single leg squats: *(3 sets × 10 reps - each side)* Resisted lateral side-steps: *(3 sets × 12 reps/step - each sides)*
**Plyometics**	Tuck jump: *(3 sets × 10 reps)*	Broad jumps: *(3 sets × 10 reps)*	180° tuck jumps: *(3 sets × 5 reps in each direction)*	90° hop turns: *(10 reps - clockwise and anti-clockwse)*	Double leg lateral jumps over mini-hurdle: *(3 sets × 10 reps)*	Single leg lateral jumps over mini-hurdle: *(3 sets × 10 reps)*
**Speed/Agility**	Figure of 8 runs: *(10 m course, 5 reps in each direction)*	Ladder: forward run through: *(10 reps)*	Ladder: lateral run through: *(10 reps - each way)*	Ladder: lateral hop through: *(10 reps - each way)*	Ladder: hopping slalom drill: *(10 reps)*	Lateral shuttle runs: *(10 m course, 2 sets × 10 reps)*

To date little or no research has been done to develop a treatment or impairment based classification system that addresses the multi-factorial nature of CAI, hence a comprehensive multi-component programme was implemented. Exercises were to be progressed from simplistic to increasingly complex, and were based on a compilation of previously published rehabilitation protocols to reflect current practices coupled with elements that addressed the functional demands of this particular individual [5-9]. Given the dynamic nature and functional demands of the athlete's sport, and his propensity to feelings of vulnerability whilst engaging in higher velocity activities, it was also deemed appropriate to incorporate a speed and agility component into the rehabilitation protocol. The athlete performed 5 exercise sessions per week. The first session of each week was undertaken under the direct supervision of the primary author (JOD), whilst the other four sessions were performed by the athlete without direct supervision.

## Results

### Cumberland Ankle Instability Tool

Following the intervention the athlete again completed the CAIT questionnaire. His post-intervention CAIT score was 27, differing from the pre-intervention score by 23 points (Table 2).

**Table 2:** Pre and post-intervention results

	Pre-intervention	Post-intervention	mSMD
**CAIT Score**	4	27	
**SEBT anterior (% leg length)**	82.27 ± 1.65	90.85 ± 3.54	3.30
**SEBT posterior-medial (% leg length)**	85.68 ± 0.53	93.12 ± 5.48	2.47
**SEBT posterior-lateral (% leg length)**	85.07 ± 0.72	93.74 ± 2.45	5.47
**Drop landing ankle joint plantar flexion at IC (**°**)**	29.76 ± 1.30	24.08 ± 2.36	3.10
**Drop vertical jump ankle joint plantar flexion at IC (**°**)**	27.88 ± 3.26	23.43 ± 4.73	1.11
**Walking vGRF (N)**	1028.22 ± 53.42	1009.66 ± 15.92	0.53
**Walking lateral GRF (N)**	69.55 ± 6.34	60.96 ± 7.83	1.21
**Walking braking GRF (N)**	190.03 ± 25.30	166.50 ± 8.47	1.39
**Rear-foot inversion at IC (**°**)**	2.16 ± 0.60	2.24 ± 0.65	0.12

mSMD = modified standardized mean difference

CAIT = Cumberland Ankle Instability Score

IC = initial contact

SEBT = Star Excursion Balance Test

vGRF = vertical ground reaction force

### Star Excursion Balance Test

Pre- and post-intervention SEBT directional reach distances along with associated modified SMD are shown in Table 2. Post-intervention, it can be seen that reach differences in all planes increased compared to pre-intervention values, indicating an improvement in dynamic postural stability. The associated modified SMD scores were all strong.

### Drop landing and drop vertical jump

As can also be seen from Table 2 the angle of ankle joint plantar flexion at initial contact during the drop landing, and the second impact of the drop vertical jump were reduced upon completion of the 6 week intervention. The associated modified SMD scores were all strong.

### Walking

Following the six week training programme there was no change in rear-foot frontal plane angular displacement (2.16 *vs *2.24). There was however a reduction in the peak vertical, lateral and braking components of ground reaction force (Table 2).

## Discussion

The primary outcome from this study was that a dynamic six-week neuromuscular training programme resulted in improvements in a number of sensorimotor insufficiencies that have been associated with CAI.

The results of the present study concur with those of McKeon et al [6] and Hale et al [7], whose studies both show an improvement in function as measured by the FADI upon completion of a 4-week rehabilitation programme. In contrast to both of these studies we chose to quantify functional ability using the CAIT. Upon reflection, this scale may not have been the most appropriate choice as the responsiveness of the CAIT to improvements in ankle joint function has yet to be established. Furthermore, the CAIT was specifically developed to identify the presence of FI and consideration must be given in the future to the concomitant presence or absence of MI in accordance with the recommendations of Delahunt et al [3]. However in spite of these short-comings, there was a large change in the athlete's CAIT score from 4 pre-intervention to 27 post-intervention indicating an improvement in ankle joint function and resolution of the symptoms associated with ankle instability. This change in function also concurs with an informal interview with the athlete 4 months following completion of the training programme, whereby the athlete indicated "... I haven't gone over on it since and functionally it feels much more stable...". The researchers have been able to keep in contact with the athlete, who has now resumed full participation in rugby union at club level. The athlete has not experience any further episodes of ankle joint giving way. Furthermore, the athlete completed the CAIT, 12 months following the completion of the intervention scoring 29 points out of a possible 30. One point was deducted from the overall score as the athlete in response to question 2 (My ankle feels UNSTABLE), responded; sometimes during sport (not every time). Thus, it can be seen that there has been a large improvement in the athlete's self-reported ankle joint function as a consequence of the intervention programme.

Decreased postural stability has been identified as a risk factor for ankle joint sprain [13,14]. It has consistently been shown in the literature that decreased dynamic postural stability is a feature of CAI [15]. Following completion of the 6-week training programme the athlete in the present study improved on all three SEBT reach directions. The post-intervention improvements are consistent with those observed by McKeon et al [6].

Aberrant ankle joint positioning during the transition from an unloaded to a loaded posture has been identified as a risk factor for ankle joint injury [10]. Specifically increased ankle joint plantar flexion upon initial contact with ground during jump landing has been hypothesized to predispose to the development of ankle joint sprain [10]. The results of the present study indicate that the 6-week dynamic neuromuscular training programme reduced the angle of ankle joint plantar flexion upon initial contact during jump landing, and the second initial ground contact during the drop vertical jump task. The 6-week neuromuscular training programme incorporated the principles of feed-forward neuromuscular control with the athlete required to perform numerous jump landing tasks. This observation is similar to that reported on in the ACL literature, which indicates that specific neuromuscular training can reduce aberrant valgus knee joint positioning during jump landing in female athletes [23]. The observed changes in ankle joint positioning in our study are likely to be a direct consequence of the training programme as the changes observed are greater than the measurement error of the system [28].

During walking no change was noted in the position of rear-foot inversion which further corroborates the findings of McKeon et al [5]. However, in the present study a decrease was observed in the vertical, lateral and braking components of GRF. A decrease of 18.56 N was observed for the vertical GRF, 8.59 N for the lateral GRF, and 23.53 N for the braking GRF. These observed post-intervention changes are quite small, but maybe important when one considers that even small changes in the magnitude, rate, and timing of loading may contribute to ankle joint articular cartilage degeneration [29].

The present study does have a number of limitations. Firstly it is a case report and thus extrapolation to all subjects with CAI requires careful consideration. Therefore, further studies with a randomized controlled design are required to fully determine the effects of dynamic neuromuscular training programmes on ankle joint sensorimotor control. Furthermore, during the pre- and post-intervention test session we did not record any lower limb electromyographic activity. This would have afforded us a greater understanding of ankle joint sensorimotor control, and in particular the initiation and magnitude of muscle activity [30]. This may be important as previous research has indicated that subjects with ankle instability have altered muscle activation patterns [21,22].

The 6-week dynamic neuromuscular training programme improved parameters of ankle joint sensorimotor control and subjective function in an athlete with CAI. Further research is now required in a larger cohort of subjects with CAI to determine the effects of neuromuscular training programmes on ankle joint injury risk factors.

## Competing interests

The authors declare that they have no competing interests.

## Authors' contributions

JOD and ED conceived and performed the study and drafted the manuscript. FK participated in the design of the study, evaluation of the data and writing the manuscript.
